# Pharmacological action of *Hedysarum* polysaccharides: a review

**DOI:** 10.3389/fphar.2023.1119224

**Published:** 2023-08-28

**Authors:** Xiang Gao, Chunzhen Ren, Linyu Li, Huilin Zhao, Kai Liu, Mengjie Zhuang, Xinfang Lv, Xiaodong Zhi, Hugang Jiang, Qilin Chen, Xinke Zhao, Yingdong Li

**Affiliations:** ^1^ School of Traditional Chinese and Western Medicine, Gansu University of Chinese Medicine, Lanzhou, China; ^2^ Gansu Province Key Laboratory of Chinese Medicine for the Prevention and Treatment of Chronic Diseases, Lanzhou, China; ^3^ Key Clinical Specialty of the National Health Commission of the People’s Republic of China, Key Specialized Cardiovascular Laboratory National Administration of Traditional Chinese Medicine, Lanzhou, China; ^4^ Affiliated Hospital of Gansu University of Chinese Medicine, Lanzhou, China; ^5^ First School of Clinical Medicine, Gansu University of Chinese Medicine, Lanzhou, China; ^6^ Xinjiang Medical University School of Basic Medicine, Urumqi, China

**Keywords:** HPS, anti-inflammatory, complications of diabetes, anti-tumor, traditional Chinese medicine

## Abstract

*Hedysarum*, a traditional Chinese herbal medicine and food with a long history of clinical application, is used to improve health conditions and treat various diseases. *Hedysarum* polysaccharides (HPS), flavonoids, saponins, and alkaloids, are the primary components of *Hedysarum*. HPS is the most important natural active ingredient of *Hedysarum*, which has many pharmacological effects. Currently, HPS exhibits significant promise in drug development for various ailments such as tumors, diabetes, cardiovascular diseases, Alzheimer’s disease, and fibrosis. This review paper discusses the extraction, separation, and content determination techniques of HPS, along with the investigation of its chemical constituents. More importantly, we reviewed the anti-inflammatory pharmacological effects of HPS, such as inhibition of inflammatory factors and NF-κB signaling pathway; antitumor activity through apoptosis induction in tumor cells and blocking tumor cell proliferation and metastasis; antioxidant effects; regulation of various cytokines and immune cells; regulation of blood sugar levels, such as in type I and type II diabetes and in diabetic complications; improvement in symptoms of Alzheimer disease; anti-aging and anti-fibrosis properties; and improvement in cerebral ischemia-reperfusion injury. This review paper establishes the theoretical foundation for future studies on the structure, mechanism, and clinical use of HPS.

## 1 Introduction

*Hedysarum polybotrys Hand.-Mazz.* is a plant that belongs to the family Leguminosae. It invigorates the qi and aids the ascending yang, stops sweating, induces diuresis and relieves swelling, promotes salivation and flow of nourishing blood, relieves arthralgia, and supports the expulsion of poison and pus (China Pharmacopoeia, 2020). HPS is the main active component of *Hedysarum*, a traditional Chinese medicine (TCM), which is extracted from the dried root of *Hedysarum* and contains active polysaccharides (Qiang et al., 2018). It has several pharmacological effects, such as anti-tumor, anti-aging, immune enhancement, and anti-inflammatory properties (Jia et al., 2020; Feng et al., 2021). In recent years, several studies have compared HPS with *Astragalus* polysaccharide (APS) and have discovered that HPS is more effective than APS in terms of immunomodulation and antioxidation (Bai et al., 2020). HPS is widely used because of its advantages of low toxicity, no residue (easy to be absorbed by the body), non-tolerance (the body’s responsiveness to the drug does not decrease after repeated administration). As a result, the utilization of HPS is gaining more attention from scholars worldwide. This paper provides an overview of the current research on the extraction, isolation, and pharmacological effects of HPS. Additionally, it analyzes and summarizes the potential value of HPS, thus, serving as a reference for further research on HPS and its application in the fields of food, health products, and pharmaceuticals.

## 2 Extraction and content determination of HPS

### 2.1 Extraction of HPS

Based on their characteristics, polysaccharides are commonly extracted using chemical methods such as water, acid, and alkali extraction, physical methods such as microwave and ultrasonic extraction, and biological methods such as enzyme extraction. Traditional water extraction and alcohol precipitation, enzymatic, and ultrasonic extraction methods have been primarily detailed in the literature.

#### 2.1.1 Water extraction and alcohol precipitation method

Water extraction and alcohol precipitation is a traditional HPS extraction method and is also the most widely used. Ma (2008) adopted the orthogonal experimental design to obtain the optimal extraction of crude polysaccharide from *Hedysarum rubra* by considering the ethanol soaking time, the ethanol reflux time, the medicinal materials-to-concentrated solution ratio as an index, and 70% ethanol-precipitated HPS content in a crude product as an index. The process involved soaking medicinal materials in ethanol for 12 h and then refluxing with ethanol for 1 h to achieve a volume ratio of 1:2 between the medicinal materials and concentrated solution. The ideal temperature and duration for the two-step water extraction and alcohol precipitation process of HPS were found to be 60°C and 1 h, respectively, as reported by Yuan et al. (2015) considered the amount of water added, warm soaking time, warm soaking temperature, and extraction time as the orthogonal test factors, the HPS yield, and the free radical scavenging rate of 1,1-Diphenyl-2-picrylhydrazyl (DPPH), which can reach 0.83%, as the indexes. After a comprehensive evaluation, the optimum water extraction and alcohol precipitation process of HPS was obtained at a temperature of 70°C, performed twice in 15 times the water volume for 3 h with alcohol.

#### 2.1.2 Microwave-assisted extraction

Liu et al. (2007) investigated the extraction of HPS using microwave-ultrasonic technology and compared it to the traditional extraction methods. The results demonstrated that the traditional approach produced a polysaccharide content of 4.19%, whereas the microwave-ultrasonic process resulted in a significantly higher concentration of 8.98%. Additionally, Zhao B. et al. (2015) employed a microwave-assisted technique to extract HPS and utilized a response surface methodology to optimize the process. The optimal extraction conditions included a 45-minute extraction period, 213 W of microwave power, 80°C, a liquid-to-material ratio of 26 to 1, and a 1-hour extraction period. Under these conditions, the HPS yield reached ∼10.11%.

#### 2.1.3 Enzymatic hydrolysis

Tan (2010) considered enzyme dosage, enzymolysis temperature, and time as orthogonal factors, with the optimum technological conditions for enzymatic extraction of HPS cellulose being: enzyme dosage of 0.3%, enzymolysis temperature of 40°C and enzymolysis time of 110 min. Wei et al. (2014), based on a uniform design and single-factor experiment, considered enzyme amount, enzymolysis time, extraction time, and water amount as experimental factors, with the HPS extraction rate and total saponins content as indexes. They optimized the enzymatic extraction process of Radix *Hedysarum* by using the quadratic general rotation combination design as indicated below: 280 mg of enzyme, 90 min of enzymolysis, water amount at 21 times, and 180 min of extraction. Enzymatic hydrolysis, a biological extraction method, has considerable development prospects because of its high specificity, small dosage requirement, and little chemical pollution.

In summary, the extraction methods, conditions, and yield of HPS are shown in Table 1.

**TABLE 1:** The extraction methods, conditions, and yield of HPS.

Solvent extraction	Liquid to material ratio (g/mL)	Solvent	Extraction times	Extraction time (min)	Free radical scavenging rate	Extraction temperature (°C)	Extraction frequency (kHz)or power (W)	HPS yield (mg/100 g)	Refrences
Water extraction	1:15	70% ethanol	2	60	0.83%	60	-	10.11	Ma, 2008; Yuan et al. (2015)
alcohol precipitation	1:2	-	2	60	-	60	-	-	Ma, 2008; Yuan et al. (2015)
Microwave-assisted extraction	1:26	-	2	60	-	80	213	10.11	Liu et al. (2007), Zhao B. et al. (2015)
Enzymatic hydrolysis	1:21	-	2	110	-	40	-	-	Tan, 2010; Wei et al. (2014)

### 2.2 Separation of HPS

Currently, there are limited studies on the separation methods of HPS. Madan (Ma, 2008) employed the step alcohol precipitation method to isolate polysaccharides 1, 2, and 3, which were further separated into two parts through gel column chromatography. Gas chromatography analysis showed that these three polysaccharides contained five monosaccharides: rhamnose, arabinose, xylose, glucose, and galactose. Yang Tao (Chen et al., 2012) obtained *Hedysarum* Polysaccharide 1 (HPS1) from Hongqi using water extraction and 30% ethanol precipitation which was then purified to yield four uniform components. Similarly, Chen Tongqiang et al. (Hui et al., 2010) conducted a series of separation and purification processes on *Hedysarum* polysaccharide 3 (HPS3) to isolate four uniform components, namely HPS3-A, HPS3-B, HPS3-C, and HPS3-D. Thin layer chromatography and gas chromatography analysis revealed that HPS3-A primarily consisted of glucose, while HPS3-B, HPS3-C, and HPS3-D were composed mainly of arabinose and galactose. Furthermore, Hui He Ping (Yu et al., 2005) reported the deproteinization of crude polysaccharide of *Hedysarum* membranaceus through the trichloroacetic acid n-butanol method, followed by purification using Sephadex G-25 column chromatography, to obtain a uniform component of *Hedysarum* membranaceus polysaccharide 2 (HPS2). HPS is a heteropolysaccharide composed of various types and numbers of monosaccharides, resulting in different HPS structures. Presently, research on the separation of HPS is progressing.

### 2.3 HPS content determination method

At present, HPS content is primarily determined through phenol-sulfuric acid colorimetry. Here, the sulfuric acid rapidly decomposes the polysaccharide to produce monosaccharide and then dehydrates it to produce sugar aldehyde derivatives so that its content can be determined by colorimetry. Research shows that this method is simple, sensitive, accurate, and reliable with good reproducibility (Wei et al., 2013; Yang and Li, 2013). Wei Shuchang et al. (Chen et al., 2012) adopted the improved differential phenol-sulfur method to quantitatively determine the crude polysaccharide content of *Hedysarum* stilbene, with glucose being the reference substance. Ouyang Yihua et al. (Ouyang and Yan, 2013) used anthrone–sulfuric acid spectrophotometry to determine HPS content and obtained satisfactory results. Li et al. (2008) measured HPS contents using high-performance liquid chromatography (HPLC) along with an evaporative light scattering detector. For this process, HPS was separated and purified using a series of techniques to obtain glucose, xylose, rhamnose, galactose, and arabinose. Moreover, the phenol–sulfuric acid colorimetry method can determine only the total polysaccharide content but not the single component of polysaccharide. When using calorimetry, the conversion factor (f) value is needed to determine HPS, which increases the experimental error and affects the experimental efficiency. The HPLC method, in contrast, can determine the single component of polysaccharide and does not require the f value to measure HPLC content; the result, moreover, is more accurate. However, its sample pretreatment is cumbersome, and total polysaccharide content cannot be determined, so this method also has some limitations. Therefore, exploring a more efficient and accurate HPS determination method is required.

## 3 Study on the chemical structure of HPS

HPS, the primary active ingredient of *Hedysarum*, is being extensively studied in recent years. HPS are heteropolysaccharides composed of monosaccharides and polysaccharide structures, with a polysaccharide content ranging from 23% to 34%. These polysaccharides consist of five monosaccharides, namely galactose, glucose, xylose, arabinose, and rhamnose (Table 2). To date, researchers have isolated and purified four HPS fractions from *Hedysarum* polysaccharide, namely *Hedysarum* polysaccharide 1 (HPS1), *Hedysarum* polysaccharide 2 (HPS2), *Hedysarum* polysaccharide 3 (HPS3), and *Hedysarum* polysaccharide 4 (HPS4) (Liu, 2007; Ma et al., 2008; Li et al., 2015a). The known structures of HPS include HPS1 (HPS1-A, HPS1-B, HPS1-C, HPS1-D), HPS3 (HPS3-A, HPS3-B, HPS3-C, HPS3-D), HPS4 (HPS4-1A, HPS4-1B, HPS4-2A), etc. However, the specific structural formula of HPS2 has not been reported in the literature.

**TABLE 2:** Monosaccharide compounds of HPS.

Monosaccharide	Molecular formula	Molecular weight	Structural formula
Rhamnose	C_6_H_12_O_5_	164.16	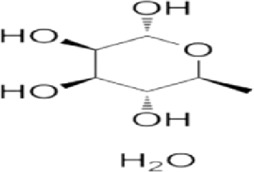
Arabinose	C_5_H_10_O_5_	150.13	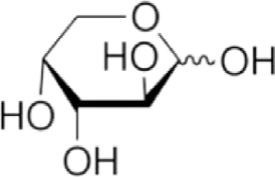
Glucose	C_6_H_12_O_6_	180.16	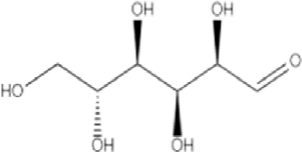
Xylose	C_5_H_10_O_5_	150.13	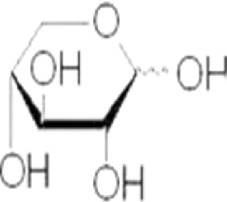
Galactose	C_6_H_12_O_6_	180.16	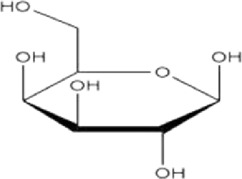

**FIGURE 1:**
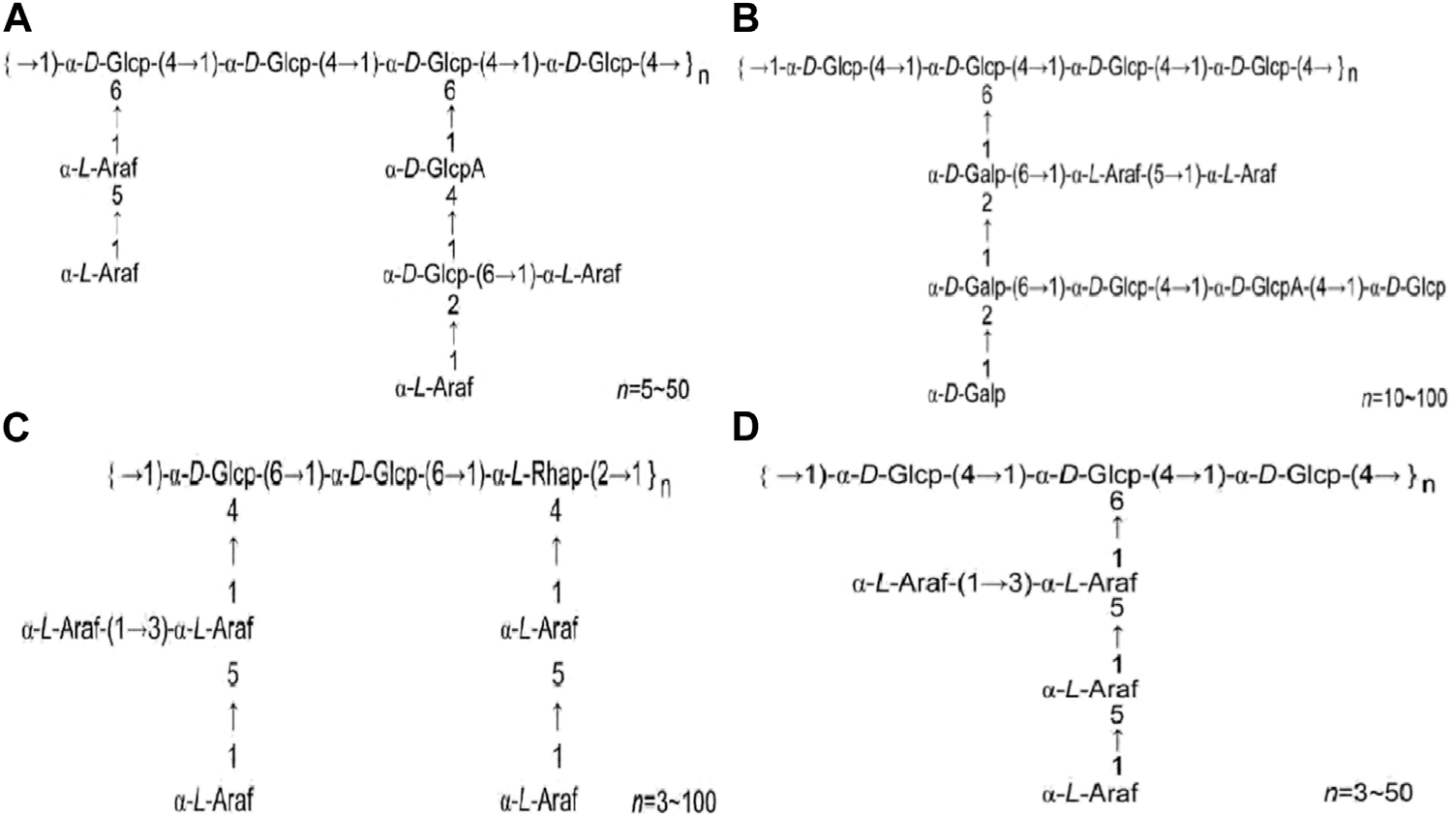
**(A)**. HPS1-A structural formula **(B)**. HPS1-B structural formula **(C)**. HPS1-C structural formula **(D)**. HPS1-D structural formula. (Chinese Journal of New Drugs, 2018, 27(19): 2271–2280.).

HPS1 has been separated and purified to yield HPS1-A, HPS1-B, HPS1-C, and HPS1-D (Feng et al., 2014a; Yang et al., 2014a; Yang, 2014). HPS1-A is primarily composed of 1, 4-α-D-Glcp and 1, 4, 6-α-D-Glcp in the main chain. HPS1-B is an acidic polysaccharide with a relative molecular weight of 5.236×10^5^, and its main chain consists of 1, 4-α-D-Glcp and 1, 4, 6-α-D-Glcp. HPS1-C contains 1, 6-α-D-Glcp, 1, 4, 6-α-D-Glcp, 1,2-α-L-Rhap, and 1, 2, 4-α-L-Rhap in the main chain. HPS1-D is a neutral heteropolysaccharide with a relative molecular weight of 4.593 × 10^4^, andits main chain consists of 1, 4-α-D-Glcp and 1, 4, 6-α-D-Glcp. Figure 1 illustrates the structural formulas of HPS1-A, HPS1-B, HPS1-C, and HPS1-D.

**FIGURE 2:**
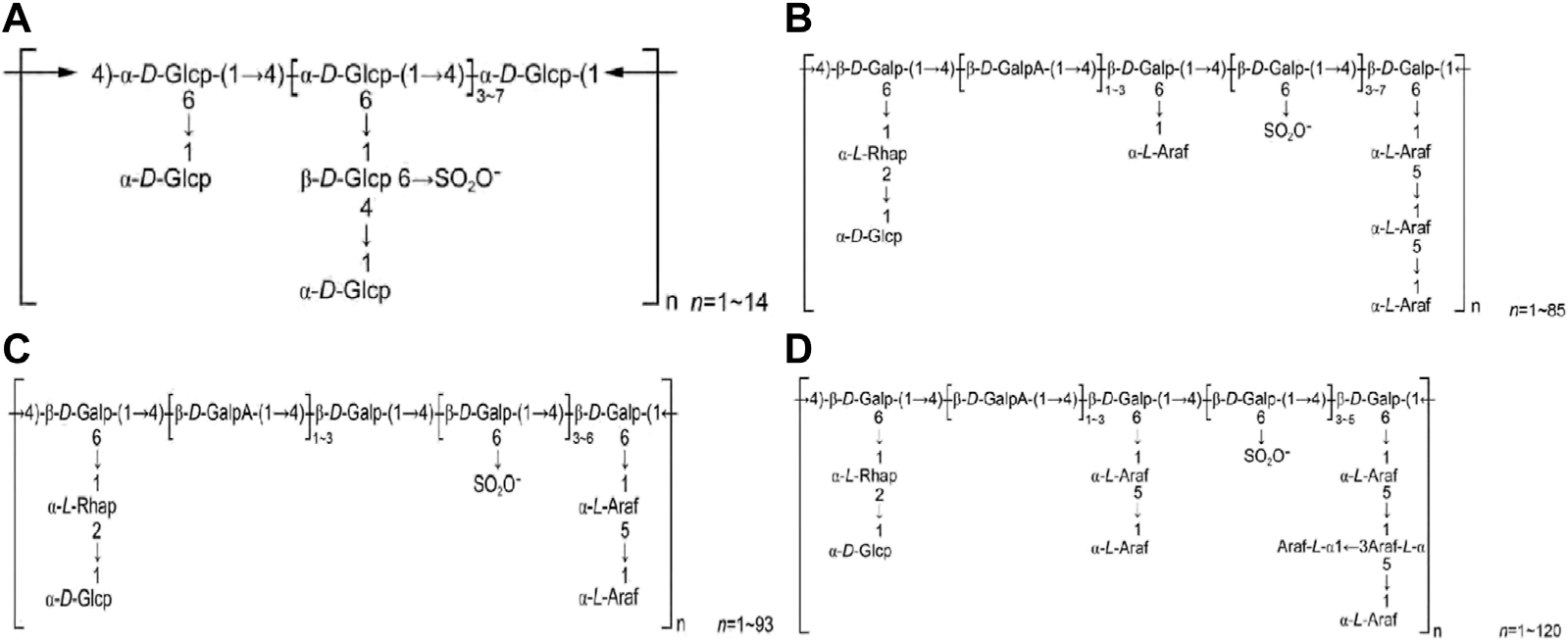
**(A)** HPS3-A structural formula. **(B)** HPS3-B structural formula. **(C)** HPS1-C structural formula. **(D)** HPS3-D structural formula (Chinese Journal of New Drugs, 2018, 27(19): 2271–2280.).

HPS2 is a heteropolysaccharide with predominantly β-glycosidic bonds, but determining precise structure requires further research. HPS3 comprises four components, namely HPS3-A, HPS3-B, HPS3-C, and HPS3-D. HPS3-A contains α-D-(1→4) glucopyranosyl group as the primary chain, while HPS3-B contains β-D-(1→4) galactose and β-D-(1→4) galacturonic acid in the main chain. HPS3-C is composed of β-D-(1→4) galactosyl and β-D-(1→4) galacturonic acid in the main chain, whereas HPS3-D consists of β-D-(1→4) galactopyranosyl and β-D-(1→4) galacturonic acid group as the main chain. The relative molecular masses of these components require further investigation. Figure 2 displays the structural formulas of HPS3-A, HPS3-B, HPS3-C, and HPS3-D.

**FIGURE 3:**
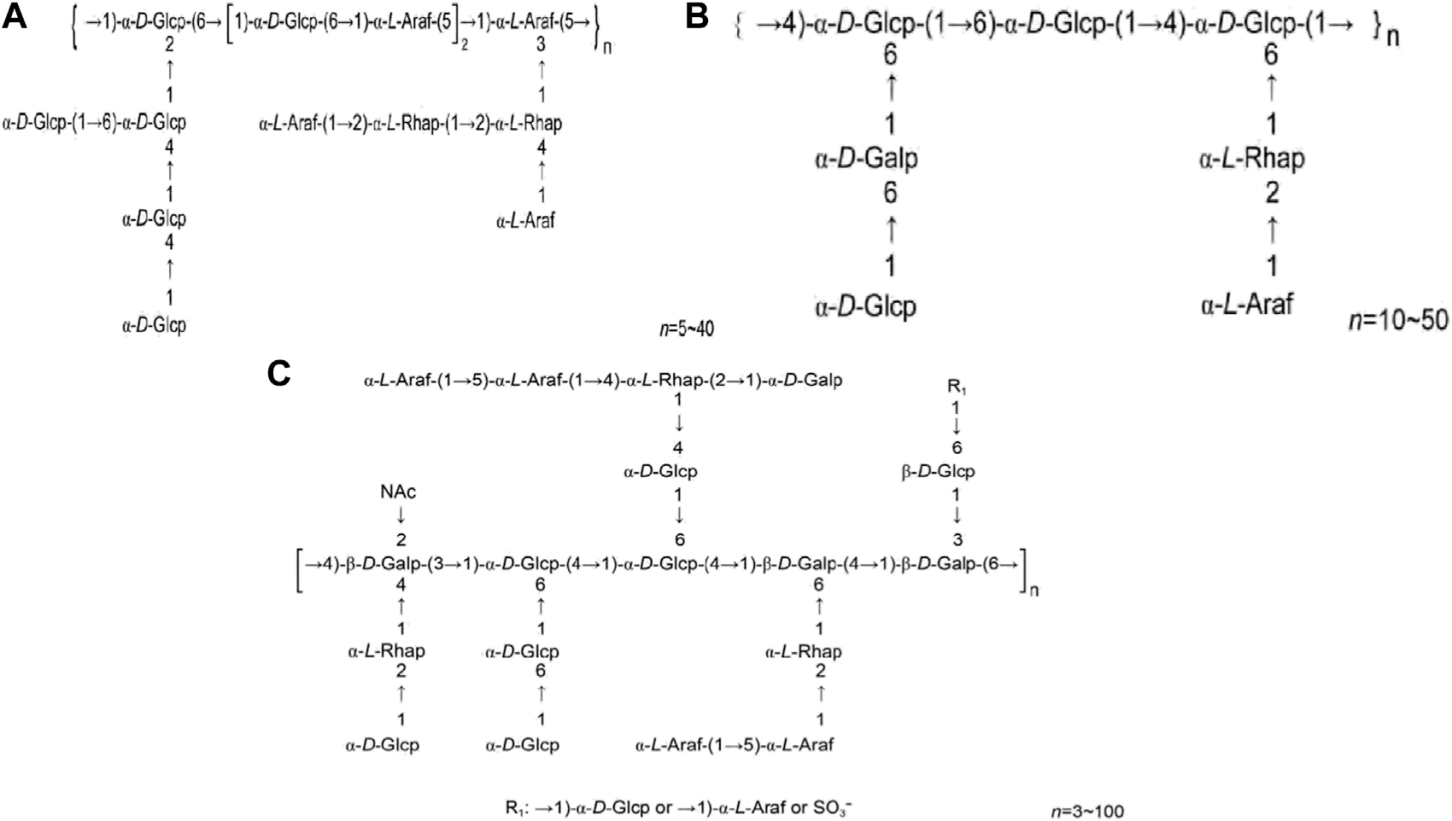
**(A)** HPS4-1A structural formula. **(B)** HPS4-1B structural formula. **(C)** HPS4-2A structural formula (Chinese Journal of New Drugs, 2018, 27(19): 2271–2280.).

HPS4 consists of three components: HPS4-1A, HPS4-1B, and HPS4-2A (Dang Z. L. et al., 2013; Dang Z et al., 2013; Feng et al., 2013). HPS4-1A is a neutral heteropolysaccharide with a relative molecular mass ranging from 7.386 × 10^4^ to 6.68 × 10^5^, and its main chain comprises 1,5 and 1, 3, 5 linked α-L-furanyl arabinose groups and 1, 6 and 1, 2, 6 linked α-D-galactopyranosyl groups. HPS4-1B has a relative molecular mass ranging from 20 to 5.023 × 10^4^, with 1, 4-α-D-Glcp and 1, 6-α-D-Galp formingthe main chain. HPS4-2A is a thioylacetylaminoamino heteropolysaccharide with a relative molecular mass of 2.72105, and its main chain consists of 1, 3,4-β-D-2-acetyl aminogalactose, 1, 4, 6-α-D-glucose, 1, 4,6-β-D-galactose, and 1, 3, 6-β-D-galactose. Figure 3 illustrates the structural formulas of HPS4–1A, HPS4-1B, and HPS4-2A.

## 4 Pharmacological action of HPS

**FIGURE 4:**
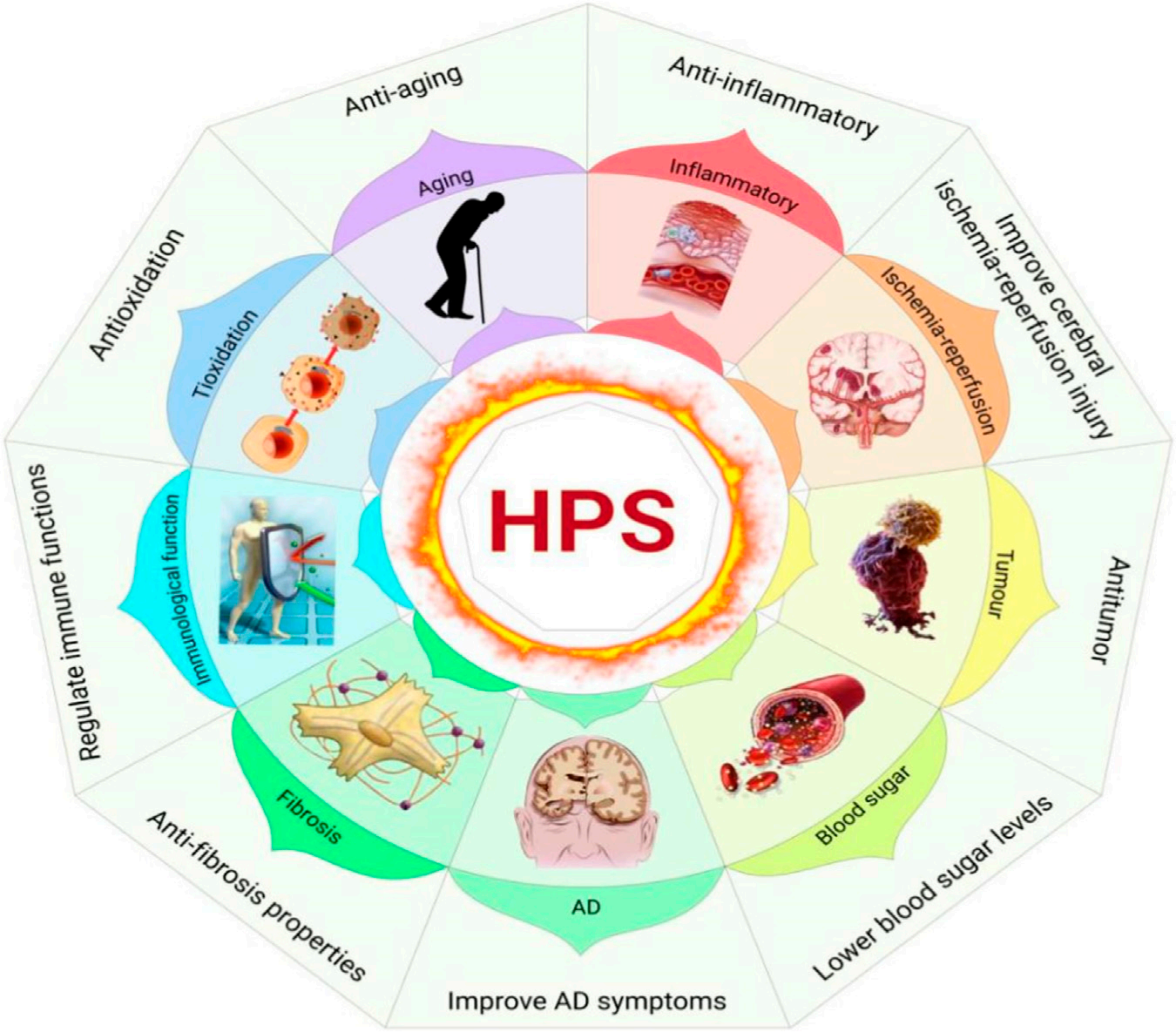
Pharmacological effects of HPS (Xiang Gao and Chunzhen Ren).

HPS has many pharmacological effects. In particular, nine of these effects have been thoroughly studied, including its anti-inflammatory, antitumor, antioxidation, anti-aging, and anti-fibrosis properties and its ability to lower blood sugar levels, regulate immune functions, improve Alzheimer’s disease (AD) symptoms, and improve cerebral ischemia-reperfusion injury (Figure 4).

### 4.1 Anti-inflammatory effect

Wang Jing et al. (Feng et al., 2014b) on studying the HPS effect on the expression of TIR-Domain-Containing Adapter-Inducing Interferon-β (TRIF) and tumor necrosis factor receptor-related factor 6 (TRAF6), a key molecule in the toll-like receptor 4 (Toll-4) signal transduction pathway in uveitis (EIU) model rats, found that intraperitoneal injection of 400 mg/kg HPS in rats lowers the mRNA expression of TRAF6 and nuclear factor-κB (NF-κB). However, it has no obvious effect on the mRNA expression of TRIF, which indicates that HPS can alleviate the endotoxin lipopolysaccharide (LPS)-induced inflammatory reaction of the anterior segment in rats by inhibiting the expression of TRAF6 nucleic acid. This provides evidence that HPS has the potential to act as a drug that inhibits inflammatory response. Shuo Yang et al. (Qiang et al., 2018) found that after intraperitoneal injection of 400 mg/kg HPS in EIU model rats, phosphorylated GSK3-β protein expression was upregulated in the iris and ciliary body, NF-κB p65 mRNA expression level was substantially suppressed, anti-inflammatory IL-10 levels in the aqueous humor were increased. In contrast, the levels of pro-inflammatory IL-6, TNF-α, and IL-1β were inhibited, indicating that HPS could inhibit LPS-induced uveitis in rats. These findings suggest that HPS has a favorable anti-inflammatory effect. Zhang et al. (Shuo et al., 2019) discovered that local joint injections of 0.36 and 0.12 mg/joint could enhance the extension range of knee joints in OA rats, decrease synovial thickness, and elevate synovial glycosaminoglycan content. Furthermore, HPS reduced the expression of inflammatory markers such as IL-6, TNF-α, and IL-1β in joint lavage fluid, indicating that HPS modulates inflammatory cells and factors to inhibit the inflammatory response. In addition, Wang Ximei et al. (Zhang et al., 2019) found that HPS (0.35 and 1.05 mg/joint) and HG-1 (0.15 and 0.45 mg/joint) can help increase the knee joint extension range in OA rats, inhibit abnormal articular surface synovial thickening, increase synovial glycosaminoglycan content, relieve free radical damage to cartilage, and improve the overall joint function in OA rats. These results suggest that HPS has the potential to be utilized for treating osteoarthritis (OA) and local inflammatory reactions in rats. In sum, HPS has satisfactory anti-inflammatory effects *in vivo*, closely related to the inhibition of IL-6, TNF-α, IL-1β, and other inflammatory cytokines.

#### 4.1.1 Inhibition of NF-κB signal pathway

**FIGURE 5:**
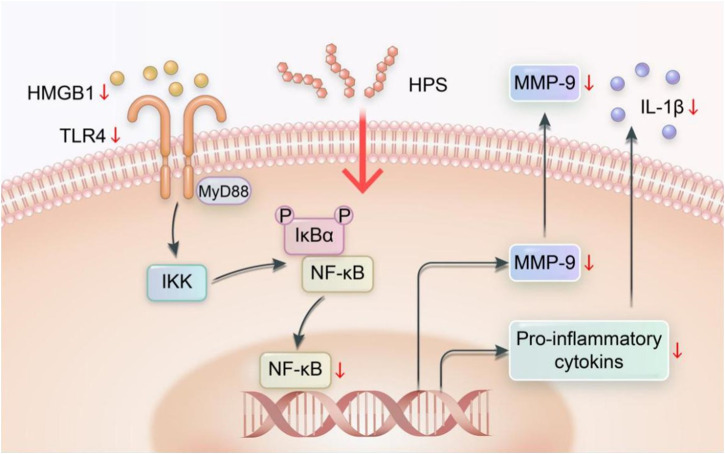
Regulation of NF-κB signaling pathway by HPS (Xiang Gao and Chunzhen Ren).

Nuclear factor-κB (NF-κB) is a transcription factor that is activated by various signals through the degradation of IκBs. Once activated, NF-κB enters the nucleus to bind with DNA (Wang et al., 2018). NF-κB activation is strongly associated with the expression of inflammatory markers and the metabolic imbalance of the extracellular matrix (ECM) (Wu et al., 2018; Xie et al., 2018). Han Weiqiang et al. (Han et al., 2019) assessed HPS-related inflammatory response in obese (ob/ob) mice with diabetic peripheral neuropathy and discovered that administering HPS in dosages of 100 and 200 mg/kg reduces NF-κB protein and mRNA expression in the nerve tissue and then that of IL-1β protein and mRNA; thus, reducing inflammation and protecting the nerves. HPS also reduces the expression of HMGB1, TLR4, NF-κB, IL-1β protein, and mRNA in the nerve tissue and reduces nerve tissue damage caused by inflammatory reaction; as a result, mice showed enhanced sciatic nerve conduction velocity and demyelination (He et al., 2019). Jin et al. (2017a) found that HPS can improve abnormal deposition of myocardial interstitial collagen in db/db mice and downregulate the expression of matrix metalloproteinase-9 (MMP-9) and NF-κB p65 and protein and mRNA in myocardial tissue. This outcome shows that HPS can aid in improving the abnormal myocardial collagen network structure, delaying the abnormal remodeling of myocardial interstitials, protecting cardiac function, and delaying myocardial interstitial fibrosis in diabetic cardiomyopathy (DCM). Inhibiting the activation of NF-κB also has a significant role in preventing the occurrence and development of diabetic retinopathy (DR) (Zhao and Liu, 2016). To sum up, the ability of HPS to inhibit NF-κB and its related proteins leads to the regulation of other protein expressions, ultimately resulting in the reduction of inflammation, delay in myocardial fibrosis, and prevention of diabetic retinopathy. HPS can reduce inflammation by suppressing the expression of NF-κB and its protein in tissues and then regulating the expression of other proteins (Figure 5).

### 4.2 Antitumor effect

HPS can prevent the formation of tumors and help treat tumors by suppressing tumor growth, stimulating cell apoptosis, and supplementing the effects of chemotherapy drugs (Zhao Y. et al., 2015). Zeng et al. (2019) found that after treating oral cancer cells with 50 μg/mL HPS for 24 h, apoptosis-related gene expression profiles, including Fas and Fas mRNA and related proteins of SCC25 were all upregulated, indicating that HPS may suppress the growth of SCC25 oral cancer cells and trigger their apoptosis via the Fas/Fasl pathway. In their study, Wang and Liu (2017) observed that treatment with 50, 100, or 200 mg/L of HPS-1 could regulate the oxidation-antioxidant capacity ratio of human lung adenocarcinoma (LUAD) A549 cells, resulting in the suppression of cell proliferation and induction of apoptosis. Additionally, it was found that HPS could attenuate the growth of LUAD A549 cells and induce apoptosis by modulating the cellular Bax/Bcl-2 ratio (Liu H. et al., 2016). Li et al. (2007a) evaluated the impact of HPS-3, 1 of the 3 components of HPS, on the proliferation and apoptosis of human gastric cancer MGC-803 cells *in vitro* by MTT assay, microfluorescence, and flow cytometry. HPS-3 at 200 g/L and 400 g/L for 72 h could induce cell apoptosis and inhibit the MGC-803 tumor cell growth and proliferation, thus, achieving an antitumor effect. The mechanism and regulation of HPS have been found to be closely associated with the modulation of Bax and Bcl-2 proteins’ expression in the mitochondria. That is, HPS can inhibit the growth of oral cancer, lung adenocarcinoma, and human gastric adenocarcinoma, thus, possessing a wide range of effects. Li et al. (2007b) isolated and purified HPS to obtain 3 components of HPS (HPS-1, HPS-2, HPS-3), with HPS-1 and HPS-3 being regarded as the most important in preventing the growth of tumor cells. In addition, the antitumor effects of HPS depend on the molecular weight and monosaccharide composition of its components. To investigate the antitumor mechanism of HPS, a group of Chinese researchers treated LUAD cell A549 with varying concentrations of HPS and observed that HPS has the ability to induce apoptosis in these cells and reduce the expression of the Bcl-2 proto-oncogene protein. To further observe the cell-cycle inhibition and apoptosis-inducing mechanism of HPS on HEP-G2 cells, the tumor cells were exposed to HPS at two distinct doses (200- and 400-µg/mL) for a total of 72 h. According to the findings, HPS exhibited the ability to inhibit the growth of human hepatoma HEP-G2 cells by inducing apoptosis, arresting cell cycle at the G2/M phase, and decreasing the expression of the proto-oncogene product Bcl-2 protein (Li and Zhang, 2009). Additionally, the administration of three HPS components, namely HPS-1, HPS-2, and HPS-3, to S180 tumor-bearing mice showed promising potential in inhibiting tumor progression and enhancing the quality of life (Li and Zhang, 2009). Tian et al. (2015) showed that HPS might suppress bladder cancer in rats, and its mechanism may involve increasing the interleukin-2 (IL-2) level in the serum, enhancing the immune function, reducing the vascular endothelial growth factor (VEGF) expression level, and inhibiting tumor angiogenesis. Using a liver cancer mouse model, Wei et al. (2011) illustrated that HPS has the potential to prevent liver cancer cells from metastasizing via the blood flow. The studies mentioned above demonstrate that HPS exerts its anti-tumor effects through various mechanisms, such as activation of the Fas/Fasl pathway and upregulation of Bax expression leading to induction of apoptosis, enhancement of immunity via increased IL-2 levels, downregulation of Bcl-2 protein expression resulting in the promotion of apoptosis, reduction of VEGF expression leading to inhibition of tumor angiogenesis, arrest of the cell cycle in the G2/M phase, and inhibition of tumor cell growth. HPS exhibits potential anti-tumor properties against lung cancer, liver cancer, and bladder cancer, as summarized in Table 3.

**TABLE 3:** Anti-tumor effect mechanism of HPS.

Pharmacological action	Function	Living mode	References
Inhibition of tumor cell, promote tumor cells apoptosis	Expression levels of both the mRNA of the genes Fas and Fas and their associated proteins were upregulated	SCC25	Zeng et al. (2019)
	Regulation of the oxidative/antioxidative capacity ratio in human A549 cells	A549 cells	Wang and Liu (2017)
	Regulation of the intracellular Bax/Bcl-2 ratio	A549 cells	Liu C. et al. (2016)
	Regulation of Bcl-2 and Bax protein expression in mitochondria	MGC-803 cells	Li et al. (2007a)
	Reduced expression of Bcl-2 protein	A549 cells	Liu C. et al. (2016)
	Blocking the progression of the G2/M phase of the cell cycle and reducing Bcl-2 protein	HEP-G2 cells	Li and Zhang (2009)
Enhances immune function and inhibits tumour angiogenesis	Increasing serum levels of IL-2. Reducing VEGF expression levels	Bladder cancer rats	Tian et al. (2015)
Inhibition of tumour cell metastasis	Inhibition of hepatocellular carcinoma cell metastasis via blood stream	Liver cancer mice	Wei et al. (2011)

### 4.3 Antioxidation

Several studies have shown that HPS and its active ingredients have unique anti-aging and antioxidation effects, and their active ingredients are mainly concentrated in polysaccharide compounds (Zhou et al., 2020). Yuan et al. (2019) adopted DPPH, superoxide anion, 2,2′-diazobis (3-ethylbenzothiazoline-6-sulfonic acid) [diammonium 2,2′-Azino-bis-(3-ethylbenzothiazoline-6-sulfonate), ABTS], and free radicals to measure the antioxidant activity of HPS. With the increase in HPS concentration, the scavenging ability of DPPH, superoxide anion, and ABTS increased, demonstrating a certain dose-effect relationship. Liu C. et al. (2016) found that HPS aqueous solution in dosages of 200, 100, and 50 μg/g showed anti-aging effects. After 42 days of intragastric administration in mice, the thymus and spleen indexes improved; this improvement helped slow down the atrophy of immune organs in aging animal models, thus, showing that through regulating T lymphocytes and increasing IL-4 levels, HPS efficiently enhanced the cellular and humoral immune capabilities in aged subacute mice. Zhao et al. (2014) adopted an anion exchange and gel permeation chromatography method to isolate a water-soluble polysaccharide (HPS3aS) measuring 1.22 × 10^4^ Da in molecular weight from the *Hedysarum* root. The scavenging experiments with 1,1-diphenyl-2-methylhydrazine, superoxide, and hydroxyl radical showed the good antioxidant activity of HPS3aS *in vitro*, implying that it is a potent antioxidant. HPS may also enhance Bcl-2 expression, suppress Bax expression, and effectively block the apoptosis of cells, thus, delaying the aging of brain cells (Shi et al., 2019). Kou et al. (2015) extracted and separated HPS components, namely, HPS-M, HPS-C, and HPS-H, using ultrasonic-assisted, microwave-assisted, and conventional hot water extraction methods, respectively. HPS-M showed higher antioxidant activity *in vitro* than HPS-C and HPS-H, and HPS-M showed a stronger ability to scavenge free oxygen radicals. The study by Yang Jie et al. investigated the effects of HPS on human brain microvascular endothelial cells under oxidative damage (Yang et al., 2016). HPS showed cellular protective effects of oxidized low-density lipoprotein, as it can potentially scavenge free radicals, inhibit apoptosis, and resist oxidative stress. To observe the effect of HPS on the scavenging rate of superoxide anion and hydroxyl radical in rats, some researchers injected 50, 100, 200, and 400 mg/kg HPS into the abdominal cavity of Wistar rats. According to Yang Jie et al., HPS was found to enhance the scavenging capacity of the superoxide anion and hydroxyl radical, as well as improve the activities of glutathione peroxidase and superoxide dismutase (SOD) (Lei et al., 2015). These studies suggest that HPS may increase the scavenging capacity of DPPH, superoxide anion, and ABTS in cells, improve immune organ atrophy, regulate T lymphocytes, and increase the level of IL-4. Additionally, HPS has been found to enhance the expression of Bcl-2, inhibit the expression of Bax, and suppress cell apoptosis. Furthermore, HPS has the potential to improve the scavenging capacity of hydroxyl radicals, and enhance the activity of glutathione peroxidase and superoxide dismutase (SOD), thus, providing resistance to oxidative stress. HPS exhibits strong antioxidant activity both *in vivo* and *in vitro*.

### 4.4 Effect of HPS on regulating blood sugar levels

Diabetes is a common endocrine and metabolic disease that causes considerable and serious damage to human health. According to the International Diabetes Federation, the number of diabetes cases worldwide is expected to increase from the current 425 million to an estimated 630 million by 2045 (Matoba et al., 2019). The HPS have a certain curative effect on diabetes, as they can help lower blood sugar levels and prevent and treat its various complications. HPS is under research for its ability to prevent and treat diseases of the kidney, myocardium, liver, retina, and nervous system caused by diabetes.

Mice with type 2 diabetes were given HPS in dosages of 50, 100, 200, 400, and 600 mg/kg for 28 days. According to Li Xiaodong et al. (Ye et al., 2020), HPS significantly improved blood lipid metabolism disorder by reducing levels of fasting blood glucose, glycosylated hemoglobin, total cholesterol, and triglycerides, while increasing high-density lipoprotein levels. Type 2 diabetes is characterized by the body’s inability to effectively use insulin. To investigate the effects of HPS on type 2 diabetes, rats were administered doses of 50, 100, and 200 mg/kg of HPS-3 by Li et al. (2012). Blood glucose, glucose tolerance, and lipid profiles were measured after 5 weeks. HPS promoted glycogen production in the rats’ livers, restored dysfunctional islet β cells, and reduced insulin resistance (IR). Oxidative stress–producing sex signal molecules can interfere with some insulin signal transduction pathways, leading to IR. Oxidative stress is closely related to inflammatory reactions.

Db/db mice were orally administered with HPS continuously for 8 weeks at doses of 200, 100, and 50 mg/kg, and the study showed the following outcomes: the peroxisome proliferator-activated receptor (PPAR) γ protein, and mRNA expression were upregulated, while that of NF-κB mRNA and protein was downregulated. HPS can potentially alleviate oxidative stress and inflammatory reaction and inhibit the progress of myocardial fibrosis caused by hyperglycemia (Zhang et al., 2017a). Wang et al. (2013) illustrated that HPS had no impact on normal mice’s blood sugar levels but may remarkably lower the blood sugar levels of the alloxan model mice and in the glucose tolerance test of normal mice and alloxan model mice, suggesting that the hypoglycemic mechanism of HPS is related to improving the body’s sensitivity to insulin. Xu (2009), using alloxan to create a Kunming mouse model of type 1 diabetes mellitus (DM), showed that HPS at different molecular weight ranges had a satisfactory hypoglycemic effect, effectively increasing the liver glycogen content. HPS also improved the thymus and spleen indexes of these DM mice, which proved beneficial in reducing damage in other tissues caused by modeling and in lowering blood sugar levels. Zhao et al. (2009) extracted, separated, and purified HPS through stepwise alcohol precipitation. The systemic symptoms of DM mice treated with HPS-1, HPS-3, and HPS-4 showed improvement, the blood sugar level was significantly reduced, and the glucose tolerance level increased. These findings provide additional evidence of the significant hypoglycemic effects of HPS on both type 1 and type 2 diabetes, which offers new possibilities for utilizing plant polysaccharides in the treatment of diabetes. The studies mentioned previously demonstrate that HPS can reduce fasting blood glucose, glycosylated hemoglobin, total cholesterol, and triglyceride levels, increase high-density lipoprotein levels, and prevent hyperglycemia-induced myocardial fibrosis. Furthermore, HPS effectively enhances the expression of PPAR γ protein, increases liver glycogen content, and improves glucose tolerance levels.

#### 4.4.1 Therapeutic effect of HPS on diabetes complications

Major vascular complications of diabetes include Diabetic nephropathy (DN), DCM, DR, nonalcoholic fatty liver disease (NAFLD), and diabetic neuropathy, with lipid-lowering and hypoglycemic therapy being the primary treatment methods used to prevent complications. HPS at different doses in DM rats can significantly improve blood glucose levels, decrease TC, TG, and LDL levels, and improve lipoprotein metabolism (Jin et al., 2004a). The available research on diabetic rat models has indicated that HPS might increase the levels of serum leptin, fat leptin, fasting blood glucose, and insulin sensitivity indices (Jin et al., 2011; Sun et al., 2014). HPS can also improve serum NO, NOS, and lipid peroxide levels in diabetic models (Jin et al., 2004b). Ding et al. (2012) showed that in type 2 diabetic rats, HPS-3 could reduce fasting blood glucose levels, improve spleen and thymus indexes, reduce the damage of high glucose levels to the immune organs of rats, and enhance the body’s antioxidant defense ability.

##### 4.4.1.1 Effect of HPS on diabetic DN

Diabetic neuropathy (DN) is a chronic complication of diabetes that not only contributes significantly to the disease but also leads to end-stage renal disease (ESRD). The pathogenesis of DN is mainly related to oxidative stress caused by hyperglycemia due to abnormal glucose and lipid metabolism (Sulaimon et al., 2020). Numerous studies by Chinese researchers have investigated the protective and therapeutic effects of HPS on diabetic neuropathy (DN). HPS can potentially slow the progression of renal interstitial fibrosis in a DN model by reducing the expression of connective tissue growth factor (CTGF) and transforming growth factor-β 1 (TGF-β 1) mRNA in the kidney (Qi et al., 2015a). Qi et al. (2015b) showed that HPS could slow down the progress of DN by inhibiting the expression of PKC protein and tissue inhibitor of matrix metalloproteinase-1 (TIMP-1) mRNA in glomerular mesangial cells or by inhibiting the overexpression of PCKα and its downstream VEGF. Jin Zhisheng (Jin et al., 2017b; Jin et al., 2017c) studied the effects of HPS at dosages of 200, 100, and 50 mg/kg on kidney tissues of db/db mice with early DN. Unlike the case in the model group, except for the low-dose HPS group, blood sugar, serum creatinine, urea nitrogen, and 24 h urine microalbumin levels in the treatment groups considerably decreased, glomerular hypertrophy reduced, mesangial area widened, and the capillary basement membrane thickened in different degrees. The protein and mRNA levels of CTGF and TGF-β1 in mouse kidney tissue were significantly reduced by HPS treatment. Additionally, HPS treatment resulted in a significant increase in both the mRNA and protein levels of MMP-2 in kidney tissue, while decreasing the levels of TIMP-1. HPS at 100 mg/kg had the most significant therapeutic effect. Thus, HPS has renal protective effects on early DN, which is worthy of further study. Thus, HPS can potentially prevent early DN and slow down neuropathy in diabetes by inhibiting the expression of CTGF, TGF-β1, PKC, TIMP-1, and VEGF proteins, increasing MMP-2 levels, and reducing serum creatinine, urea nitrogen, and 24-hour urine microalbumin levels. These findings suggest that further research is needed to explore the therapeutic potential of HPS in the management of DN.

##### 4.4.1.2 Effect of HPS on DCM

DCM, a cardiovascular complication of DM, has no specific treatment protocol. The primary mechanisms causing DCM, extracellular matrix remodeling, and myocardial fibrosis include oxidative stress and inflammatory responses caused by glucose and lipid metabolism disorder. HPS can help delay DCM onset and other cardiac protection (Wang et al., 2017; Dong et al., 2018). Recent studies have indicated that the TGF-β1/Smads signaling pathway is involved in the ability of HPS to decrease myocardial fibrosis in an animal model of DCM (Jin et al., 2017d). He et al. (2017) showed that HPS upregulated PPAR by reducing blood glucose and lipid-γ levels. The progression of DCM can be delayed by increasing GLUT-4 expression. HPS has been shown to mitigate the apoptosis of human umbilical vein endothelial cells (HUVEC) caused by high glucose levels, according to Liu et al. (2012). Dong (2018) found that in the DCM model, HPS treatment increased B-cell lymphoma/leukemia-2 (Bcl-2) expression in db/db mouse myocardium tissue while decreasing caspase-3 and Bax expression. HPS has been shown to increase TGF-β1/Smads, GLUT-4, and Bcl-2 expression while decreasing the expression of caspase-3 and Bax, thereby alleviating diabetes cardiomyopathy.

##### 4.4.1.3 Effect of HPS on DR

DR is another important complication of diabetes, which can lead to blindness in patients with diabetes. HPS can potentially inhibit neovascularization and proliferation in DR by elevatingTSP-1 expression level and reducing the platelet-derived growth factor (PDGF)-B expression level in the retina of diabetic rats to protect the retina (Zhang et al., 2017b). HPS can also inhibit neovascularization and proliferation through the VFGF in the retina to prevent DR (Ru et al., 2015; Zhang et al., 2016; Shan et al., 2017). HPS reduces neovascularization and proliferation by promoting the expression of TSP-1 and PDGF-B in the diabetic retina and explains its mechanism of action (Zhang, 2018). Li (2019) administered 30 μg/g of streptozotocin intraperitoneally twice to normal rats to induce a diabetic model. After 12 weeks of treatment, the retina thickness and cell organization of the HPS group improved. The levels of pro-apoptotic factors (Bcl-2x), nuclear factor E2-related factor (Nrf2), and SOD levels were increased in the retinal tissue of the HPS group. In contrast, the levels of apoptotic factors Bax and inducible nitric oxide synthase (iNOS) were decreased. The Bcl-2/Bax positive cell ratio was significantly higher in the HPS group than in the diabetic model group. HPS can activate the Nrf2 pathway, promote the production of the antioxidant enzyme SOD, reduce the iNOS content, reduce the apoptosis of retinal tissue, and protect the retinal tissue. HPS has been shown to increase the expression levels of TSP-1, Bcl-2x, Nrf2, and SOD, while decreasing the expression levels of Bax, iNOS, PDGF-B, and VEGF, thus, delaying the onset of DR.

##### 4.4.1.4 Effect of HPS on diabetes NAFLD

Nonalcoholic fatty liver disease (NAFLD) refers to a range of liver conditions caused by metabolic stress and strongly associated with insulin resistance, including nonalcoholic simple fatty liver, nonalcoholic fatty liver hepatitis, liver cirrhosis, and hepatocellular carcinoma (Fan and Farrell, 2009). The incidence of NAFLD is as high as 70%–90% in patients who are obese and have type 2 diabetes (Kirpich et al., 2015; Tokita et al., 2017). Shang Hongxia et al. (Shang et al., 2014) proved that HPS could reduce the serum levels of low-density lipoprotein cholesterol (LDL-C), aspartate aminotransferase (AST), triacylglycerol, and alanine aminotransferase (ALT) in NAFLD rats but increases the levels of high-density lipoprotein cholesterol (HDL-C) and the expression of SOD-1 gene in the liver tissue, as well as regulates the lipid metabolism disorder in NAFLD rats. Sun et al. (2014) evaluated Sprague-Dawley rats with NAFLD caused by a high-fat diet. HPS can upregulate the PPAR by phosphorylating AMP-activated protein kinase (AMPK)-α and PPAR-α. However, sterol regulatory element binding protein-1c (SREBP-1c) was downregulated to reduce liver fat production and increase lipid decomposition, thus, improving lipid metabolism disorder. HPS has been shown to have potential in treating NAFLD by lowering LDL-C, AST, ALT, and SREBP-1c levels while increasing HDL-C, SOD-1, phosphorylated AMP, and AMPK-α levels. Additionally, HPS may increase PPAR-α expression, which can further improve NAFLD.

##### 4.4.1.5 Effect of HPS on diabetic neuropathy

The lifetime incidence of diabetic neuropathy is about 45% in T2D and 54%–59% in T1D (Feldman et al., 2019). Hyperglycemia is the most important factor causing peripheral diabetic neuropathy, but its pathogenesis remains unclear (Alam, 2020). At present, most theories tend to explain diabetic neuropathy through chronic glucose metabolism disorder and neuromicrovascular ischemia. In addition, the changes in neurotrophic factors, inflammation, and oxidative stress caused by high glucose levels, Schwann cell apoptosis, and immune response also perform an integral function in diabetic peripheral neuropathy (Hinder et al., 2012; Vincent et al., 2013). Ji et al. (2019) illustrated that HPS could lower the severity of nerve tissue fibrosis in peripheral diabetic neuropathy in ob/ob mice, with its mechanism being the elevation in the activity of antioxidant enzymes *in vivo* by upregulating Nrf2 and downregulating the expression of Keap1 protein and gene. Han et al. (2019) showed that HPS could increase sciatic nerve conduction velocity in ob/ob mice with diabetic peripheral neuropathy, decrease the expression level of NF-κB and IL-1β protein and gene, and reduce the inflammatory reaction of sciatic nerve tissue. In summary, HPS has been shown to improve the symptoms of diabetic neuropathy by upregulating Nrf2 and downregulating Keap1 protein, as well as inhibiting NF-κB and IL-1β expression.

### 4.5 Immunomodulatory effect

Yan et al. (2017) administered 100 mg/kg/d HPS aqueous solution in immunosuppressive model rats for 7 days and found an increase in the peripheral blood hemogram count, IL-2, IL-4, interferon-γ (IFN-γ), immunoglobulin-G (IgG), IgM, IgA, and immune cytokine levels, and the thymus and spleen indexes. Combining complex enzymes with ultrasonic technology, Yang et al. (2019) extracted and isolated HPS-MC, HPS-MC (50%), and HPS-MC (80%). Under cyclophosphamide-induced immunosuppressive conditions, HPS-MCs, particularly HPS-MC (80%), exhibited potent immunoregulatory effects. HPS can help improve nonspecific immune functions, specific humoral immune functions, and cellular immune functions in cyclophosphamide-induced immunocompromised mice (Shao et al., 2017a) and comprehensively and significantly regulate all immune indexes. Studies have shown that HPS can enhance immune function by increasing peripheral blood count, IL-2, IL-4, and interferon-γ levels, as well as the levels of IgG, IgM, IgA, and immune cytokines, and the indexes of thymus and spleen.

#### 4.5.1 Regulation of various cytokines

In the late 18th century, Roberts (Fennen et al., 2016), in the process of inducing rat fibroblasts to proliferate, discovered and then isolated TGF-β, a polypeptide protein. TGF-β, extensively found in tissues and transformed cells, helps regulate the growth, differentiation, and immune functions of cells (Guo and Sun, 2020). Jin et al. (2017d) found that HPS injected in DCM mice helped improve left ventricular myocardial remodeling and myocardial collagen deposition, inhibit the release of TGF-β1 and Smad2, Smad3 protein and mRNA expression in myocardial tissue, and regulate abnormal structure and function of the heart, finally delaying the progress of myocardial fibrosis. Numerous cytokines, such as PDGF and VEGF, are important factors affecting angiogenesis, inducing the myofibroblast-like phenotype of cells and helping prevent and treat vascular diseases (Siedlecki et al., 2018; Shamsdin et al., 2019). Shan et al. (2017) illustrated that intragastric administration of HPS in dosages of 50, 100, and 150 mg/kg reduces serum C-reactive protein (CRP) levels, alleviates inflammatory reactions, reduces VEGF levels, increases pigment epithelial cell-derived factor (PEDF) levels in the retinal tissue, and inhibits retinal neovascularization, protecting the retina. In addition, Zhang et al. (2017b) discovered that HPS in different doses caused the enhanced expression of thrombospondin-1(TSP-l) and the attenuated expression of PDGF-B to inhibit angiogenesis and proliferation in DR rats. Therefore, HPS can regulate various cytokines, such as inhibiting TGF-β1, Smad2, Smad3 protein, CRP, and VEGF levels, and increasing the expression of PDGF and TSP-1. This regulation is beneficial in the prevention and treatment of diabetes complications, as shown in Table 4.

**TABLE 4:** Effects of HPS on cytokines.

Cytokine	Influence	Model	References
IL-2	Upregulation	*In vivo*	Yan et al. (2017)
IL-4	Upregulation	*In vivo*	Yan et al. (2017)
IFN-γ	Upregulation	*In vivo*	Yan et al. (2017)
TNF-α	Downregulation	*In vivo*	Yan et al. (2017)
TGF-β1	Downregulation	*In vivo*	Jin et al. (2017d)
VEGF	Downregulation	*In vivo*	Shan et al. (2017)
CRP	Downregulation	*In vivo*	Shan et al. (2017)
PEDF	Upregulation	*In vivo*	Shan et al. (2017)
TSP-l	Upregulation	*In vivo*	Zhang et al. (2017b)
PDGF-B	Downregulation	*In vivo*	Zhang et al. (2017b)

#### 4.5.2 Regulation of immune cells

Early studies have shown that HPS administered in mice promotes lymphocyte transformation and has an immunomodulatory effect (Mao and Wang, 1989). Sun et al. (1994) found that HPS has both immunomodulatory and anti-aging effects on mouse cells. Researchers administered HPS-1, HPS-2, and HPS-3 to S180 tumor-bearing mice for 14 days to observe their regulating effect on the immune system. Blood, spleen, thymus, and tumor were excised for further study. HPS increased the thymus and spleen indexes, improved lymphocyte transformation function, and increased NK and LAK cell activity (Shao et al., 2017a). Li et al. (2015b) conducted an assessment of the impact of HPS on T cells and reported differential protein dot expression of HSP-3 on mouse spleen cells. HPS-3 was found to enhance the proliferation ability of T lymphocytes and IL-2 secretion. It was also observed that HPS may have influenced the protein expression in mouse spleen cells. Wan (2012) illustrated that HPS improved spleen deficiency in rats, increasing CD3^+^ T and CD4^+^ T cell counts. Subsequent studies have also shown that HPS can regulate immunity, enhance immunity, and increase the T lymphocyte subset levels (Shao et al., 2017b). Several research studies have reported that there is a dose-response pattern and variability in the anti-complement activity of individual HPS components (Yang et al., 2014b). Additionally, these studies further support that HPS has immunoregulatory effects and can promote lymphocyte transformation and proliferation, increase the activity of NK and LAK cells, enhance IL-2 secretion, and increase the count of CD3^+^T and CD4^+^T cells as well as the index of thymus and spleen. Consequently, HPS has a promising application potential for treating immunosuppressive disorders and warrants further development and application.

### 4.6 Effect of HPS on Alzheimer’s syndrome

AD is a type of senile dementia, with its primary clinical manifestation being the gradual decline of cognitive and life functions. Commonly used therapeutic drugs are *N*-methyl-d-aspartate receptor (NMDAR) antagonists and acetylcholinesterase (AChE) inhibitors, but their effectiveness is not satisfactory. In China, because of the known curative effects of TCM, these have been applied to treat AD for hundreds of years. Jewel (2015) confirmed that the protective effect of HPS on the AD cell model is related to its ability to regulate the expression of choline kinase A, Rag regulatory protein complex LAM-TOR1, transcription factor protein AP-2-δ, and ferritin. In addition, to confirm the same mechanism, Chloe et al. (2018) added HPS to SH-SY5Y cells with a final concentration of 20 μg/mL for pre-incubation for 4 h and then added Aβ1–42 with a final concentration of 5 µM to continue incubation for 48 h and then extracted total cell proteins. HPS and Se-HPS3 have been shown to have neuroprotective effects against oxidative stress and apoptosis in SH-SY5Y human neuroblastoma cells induced by Aβ25-35 and can potentially prevent or treat neurodegenerative diseases (Wei et al., 2015). Ling (2018) established an Alzheimer’s disease cell model in PC12 cells using Aβ as a stimulus. Their research found that HPS protected PC12 cells by upregulating the expression of PRKCB, which inhibited AP25–35-induced apoptosis of PC12 cells by regulating the mitochondrial apoptosis pathway through ERK1/2 signaling. The aforementioned research results provide data support for the research and development of new HPS drugs and their derivatives.

The aforementioned research findings indicate that HPS modulates various proteins, such as choline kinase A, LAM-TOR1, AP-2-δ, and ferritin, while also upregulating PRKCB expression. Moreover, the ERK1/2 signal-mediated PRKCB has a therapeutic effect on Alzheimer’s disease.

### 4.7 Anti-aging

DNA methylation, changes in telomere and telomerase, abnormal free radicals, and abnormal regulation of nervous, endocrine, and immune systems are factors responsible for accelerating the aging process (Xie and Meng, 2018). HPS has anti-free radical damage and anti-aging impacts. Huang et al. (1992) proved that HPS could prolong the lifespan of *Drosophila melanogaster*, improve the nonspecific resistance of aged mice to harmful stimuli, reduce lipid peroxide levels in plasma, and reduce lipofuscin formation. It is further proved that HPS can increase the SOD content of red blood cells and serum cortisol and testosterone levels in aged rats. Shi et al. (2019) found that the content of antioxidant GSH-Px and SOD in HPS increased, and the content of pro-oxidant MDA and MAO decreased, confirming that HPS had both antioxidant and anti-aging impacts. HPS has the potential to slow down the aging of brain cells by increasing the expression of Bcl-2, reducing Bax expression, and preventing cell apoptosis. This anti-aging effect of HPS is further supported by the aforementioned studies. Moreover, the studies suggest that HPS exhibits anti-aging properties by decreasing lipid peroxide levels, reducing lipofuscin, MDA, and MAO content, and increasing serum cortisol and testosterone, as well as GSH-Px and SOD levels.

### 4.8 Anti-fibrosis

HPS can significantly improve liver fibrosis caused by carbon tetrachloride in mice and also considerably improve bone loss caused by liver fibrosis (Chen et al., 2016). Wang et al. (2022) studied the anti-pulmonary fibrosis effect of different extracts of *Hedysarum*. A rat model of pulmonary fibrosis was created by administering bleomycin through a laryngoscope, after which the rats were given different drugs for intervention. HPS, flavonoids, and saponins were found to inhibit the proliferation and deposition of collagen fibers and improve the pulmonary interstitial fibrosis and alveolar inflammatory reaction in the rats. Han et al. (2013) proved that HPS could reduce levels of serum ALT, AST, laminin (LN), type III procollagen (PC III), hyaluronic acid (HA), type IV collagen (IV-C), and hydroxyproline (Hyp), as well as the number of collagen fibers in liver fiber model rats. HPS has demonstrated its potential as an anti-fibrotic agent in rats by inhibiting the proliferation and deposition of collagen fibers, reducing their number, and improving pulmonary interstitial fibrosis and alveolar inflammatory reaction. Moreover, it was found to decrease the levels of serum ALT, AST, LN, PC III, HA, IV-C, and Hyp.

### 4.9 Improvement in cerebral ischemia-reperfusion injury

Shao et al. (2018) found that anticipatory administration of HPS in rats enhances the antioxidant function of the brain tissue and the self-repair ability of damaged brain tissue and alleviates abnormal changes in the blood flow, thus, reducing the brain tissue and nerve function damage caused by acute focal cerebral ischemia and achieving the protective effect. Geng Geng et al. (2020) confirmed the protective function of HPS on the hippocampus of rats with cerebral ischemia-reperfusion injury, and IL-6, TNF-α, and IL-10 levels in the brain tissue of rats significantly decreased after administration of 200 mg/kg of HPS for 10 days. Anti-inflammatory factors are secreted under induction by HPS, which aids in the regulation of both pro-and anti-inflammatory factors, thus, reducing the inflammatory reaction of cerebral ischemia-reperfusion injury and exerting its protective effect. HPS has been shown to improve the antioxidant function and self-repairing ability of brain tissue, decrease abnormal changes in blood flow, lower levels of pro-inflammatory cytokines such as IL-6 and TNF-α, increase the level of the anti-inflammatory cytokine IL-10, and regulate the balance between pro-inflammatory and anti-inflammatory factors; thus, providing a potential therapeutic effect for cerebral ischemia-reperfusion injury.

## 5 Summary and research prospect

HPS is the primary active ingredient of *Hedysarum*, a traditional Chinese medicine possessing multi-component and multi-target characteristics. Its pharmacological activities are wide-ranging, playing an irreplaceable role in the treatment of modern diseases. Nine main pharmacological effects of HPS have been detailed in this study, namely, inhibition of inflammatory reaction, immunomodulation, antioxidation, anti-aging, antitumor, lowering blood sugar levels, preventing and treating AD, lowering blood lipid levels, anti-fibrosis, improving cerebral ischemia-reperfusion injury. HPS promotes the repair and regulation of the immune system. Among the several anti-aging effects of HPS, its antioxidation mechanism is the most important one; HPS is also effective in preventing and treating cancer. HPS has been shown to exhibit antitumor properties by suppressing the growth of tumor cells and promoting tumor cell apoptosis. In addition, HPS has potential therapeutic applications for managing diabetes and associated complications. HPS has been found to significantly decrease levels of blood glucose, TC, TG, LDL, NO, NOS, and lipid peroxide, while also improving lipoprotein metabolism. Moreover, HPS has been shown to increase serum leptin and fat leptin levels, fasting blood glucose, and insulin sensitivity index. HPS has also been found to enhance the spleen and thymus index, thereby providing a potential treatment option for diabetes complications. HPS, as a natural medicine, has almost no toxic or side effects and is suitable for long-term use by patients with chronic diseases. Therefore, HPS has great potential in treating diseases.

However, there are some shortcomings in the current research situation. (1) Whether it is *Hedysarum* monomer or its compound, clinical application research is still lacking. (2) Except on HPS, studies on the pharmacological effects of isoflavones from *Hedysarum* stilbene are few. (3) At present, research on the effect of HPS has been conducted on only cells and animals, and its specific pharmacodynamic material basis remains unclear. (4) Only a few reports exist on the structural modification of HPS. Therefore, future studies should expand the research scope and depth of HPS, explore whether different HPS modifiers can enhance its immunomodulatory function, and further determine the pharmacological effects of *Hedysarum* active ingredients from the classic prescription. The biological activity of more structural modifiers of HPS and the efficacy comparison of different administration methods need to be explored. The mechanisms of its effects of reducing blood lipid levels, resisting fibrosis, and preventing radiation need to be clarified. Moreover, although HPS exerts various pharmacological effects, the precise control of its dosage needs further study.

HPS is a natural compound. Currently, the main challenge in HPS research is to identify its specific components and determine its precise targets. It is important to accurately detect the nine pharmacological targets of HPS and demonstrate the remarkable effect of multi-target integration in traditional Chinese medicine. This can help fill the gaps in the research of pharmacological effects from the perspective of HPS and provide significant theoretical support for the clinical management of illnesses. It can also provide direction for further research on new ways of treating diseases with traditional Chinese medicine.
